# Children, young people and parent engagement in health intervention design and implementation: A scoping review

**DOI:** 10.1111/hex.13572

**Published:** 2022-11-08

**Authors:** Daniel Crowther, Holly McCulloch, Helen Wong, Rebecca Mackay, Catie Johnson, Jill Chorney, Krista Ritchie, Logan Lawrence, Andrea Bishop, Melissa Helwig, Janet Curran

**Affiliations:** ^1^ Strengthening Transitions in Care Lab IWK Health Halifax Nova Scotia Canada; ^2^ Faculty of Health Dalhousie University Halifax Nova Scotia Canada; ^3^ Department of Psychiatry I Department of Psychology and Neuroscience Dalhousie University Halifax Nova Scotia Canada; ^4^ Faculty of Education Mount Saint Vincent University Halifax Nova Scotia Canada; ^5^ Research and Innovation Nova Scotia Health Halifax Nova Scotia Canada; ^6^ Policy Development and Research Nova Scotia College of Pharmacists Halifax Nova Scotia Canada; ^7^ Research & Scholarly Communications Dalhousie University Halifax Nova Scotia Canada

**Keywords:** children, engagement, health interventions, parent, young people

## Abstract

**Introduction:**

Engaging children and young people (CYP) with and without their parents in health research has the potential to improve the development and implementation of health interventions. However, to our knowledge, the scope of engagement activities used with this population and barriers to their engagement is unknown. The objective of this review was to identify and describe CYP engagement with and without their parents in the development and/or implementation of health interventions.

**Methods:**

This scoping review included any primary research studies reporting on engaging CYP, with or without parents, in the design and/or implementation of health interventions. Healthcare professionals had to be involved over the course of the study and the study had to take place in either community, primary or tertiary care settings. The following databases were searched in May 2017, May 2020 and June 2021: Medline (OVID), CINAHL (EBSCO) and Embase (Elsevier). Two independent reviewers screened titles, abstracts and full‐text articles and used a previously piloted extraction form to extract and summarize information from the included articles.

**Results:**

Twenty‐eight articles discussing twenty‐four studies were included. CYP engagement throughout the research cycle was limited. There were no observed differences in the reported presence of engagement, types of interventions or outcomes of engagement between studies engaging CYP or CYP and parents. Studies engaging CYP and parents contained limited information on how these relationships affected outcomes of engagement. Engagement was enabled primarily by the maintenance of resources and relationships among stakeholders.

**Conclusions:**

Although CYP engagement often influenced health intervention and implementation design, they are inconsistently engaged across the research cycle. It is unclear whether parental involvement enhances CYP engagement. Future research should consider reporting guidelines to clarify the level of CYP and/or parent engagement, and enhance CYP engagement by fostering synergistic and sustainable partnerships with key stakeholders.

**Patient or Public Contribution:**

A parent partner with codesign experience contributed to the creation of the research questions, screened titles, abstracts and full texts, helped with data extraction and provided feedback on the manuscript.

## 1 INTRODUCTION

Patient engagement in health research refers to a collaborative relationship between patients and researchers, where patients with lived experience are actively involved in health research decisions.^1^, ^2^ Emerging evidence has shown that patient engagement is a key practice for successful health research.^1^ Patient input has the potential to improve the overall quality of outcomes and uptake of new knowledge.^3^, ^4^ As a result, several initiatives have emerged to encourage patient‐oriented research in North America and Europe.^1^, ^5^, ^6^ Although patient engagement in health research has gained momentum over the past decade, we continue to strive for a greater understanding of how engagement occurs throughout all phases of the research process, more formal evaluations of engagement activities and stronger data to support the value of partnering with these stakeholders.^7^, ^8^

The abilities of children and young people (CYP) (0–24 years of age)^9^ to participate in paediatric health research topics has been acknowledged in the literature over the past decade.^10^, ^11^, ^12^, ^13^ While there has been a shift in the methodological approach towards transforming CYP into active research partners, further research is required to determine how best to engage and involve CYP in actual practice.^14^ Current evidence demonstrates the benefits of involving both CYP and parents as important stakeholder partners in health research. Three scoping reviews have separately described the engagement process along with the associated benefits and challenges of working with either CYP, parents or both in engagement approaches.^14^, ^15^, ^16^ Together, these reviews provide a broad range of evidence related to engagement of CYP and parents in health research.

However, to our knowledge, there remains a gap in describing the scope of literature related to the health research subfield of CYP and parent engagement in the development, design and/or implementation of health interventions. Neither does there appear to be a synthesis of evidence describing differing engagement practices between CYP engagement with and without parents. Building on previous work, this review aimed to provide a comprehensive and systematic overview of published literature on CYP engagement in health research with a specific focus on intervention design and/or implementation in the presence and absence of parents. The following research questions were addressed:

1. How does CYP engagement in health intervention design and/or implementation differ with and without parental involvement?
2. What are the characteristics of the studies and health interventions that engage CYP with and without parental involvement in the design and/or implementation of the interventions?
3. How are engagement outcomes reported, including the enablers and barriers of engaging CYP with and without parental involvement in the design and/or implementation of health interventions?

## 2 METHODS

This review was conducted in accordance with the JBI methodology for scoping reviews.^17^ A integrated knowledge translation approach was used.^18^ While there is no published protocol for this review, an a priori protocol was developed by the research team.

### 2.1 Inclusion criteria

#### 2.1.1 Participants

This review considered studies that involved CYP who were between the ages of 0 and 24 years^9^ and were engaged in the codesign and/or implementation of a health intervention. Parental involvement in the codesign and/or implementation process was not a requirement for inclusion; however, if parents were involved, the article was included. Articles that engaged only the parents were excluded.

#### 2.1.2 Concept

Any health intervention and/or implementation strategy—including programmes, tools or frameworks—that were codesigned with CYP to improve any facet of CYP health were included. Informed by patient engagement hierarchies,^19^, ^20^ we defined engagement as CYP or CYP and parents who were consulted and informed about the research project and were directly involved in decisions related to the design of intervention and/or implementation components. Interventions that did not target CYP health outcomes (i.e., targeted parent health outcomes) and that were delivered by non‐health care providers (i.e., teachers) were excluded. Articles that reported outcomes, including qualitative outcomes, related to the process of CYP engagement and health outcomes of intervention were included. Articles without any reported outcome measures related to the process of engagement were excluded.

#### 2.1.3 Context

Community, primary and tertiary healthcare settings were considered for this review.

### 2.2 Types of sources

All primary research study types were considered for inclusion. Experimental and quasi‐experimental study designs, including randomized‐controlled trials, nonrandomized‐controlled trials, pre–post trials and interrupted time series, were considered. While examining health intervention efficacy was beyond the scope of this review, these types of study designs were included in the event that study authors reported details relating to the engagement of CYP or CYP and parents in intervention design and/or implementation. Observational studies including prospective and retrospective cohort studies, case–control studies and cross‐sectional studies were considered. Qualitative and mixed‐methods studies were also considered. Systematic reviews and meta‐analyses were not included; however, relevant evidence syntheses identified in our search were reviewed for relevant articles. Text, commentary and opinion articles were excluded.

### 2.3 Search strategy

The research team established search parameters in partnership with a library scientist. A mix of controlled vocabulary such as Medical Subject Headings or Emtree terms was used in combination with keywords. The search strategy, including all identified keywords and index terms, was peer‐reviewed by a JBI‐trained information specialist, and was adapted for each included database and information source (Supporting Information Appendix: Tables [Link], [Link], [Link]). No date limit was set for the included articles.

### 2.4 Information sources

The databases searched include Medline (OVID), CINAHL (EBSCO) and Embase (Elsevier). Database searches were conducted on 29 May 29 2017, 22 May 2020 and 23 June 2021. A manual search of the table of contents from the last 5 years was also conducted for the following relevant journals: *Implementation Science*, *Journal of Pediatrics*, *BMC Health Services Research and Paediatrics* and *Child Health*. Given the range and breadth of primary sources identified through our search of the published literature, our team was not confident that a grey literature search would yield significant value to warrant expenditure of our limited resources.

### 2.5 Study/source of evidence selection

Following the search, all identified citations were collated and uploaded into Covidence systematic review software (Veritas Health Innovation) and duplicates were removed. Titles and abstracts were screened by two or more independent reviewers for assessment against the inclusion criteria for the review. Potentially relevant sources were retrieved in full, and their citation details were imported into Covidence systematic review software. The full text of selected citations was assessed in detail against the inclusion criteria by two or more independent reviewers. Reasons for exclusion of sources of evidence at full text that did not fulfil the inclusion criteria were recorded. Any disagreements between the reviewers at each phase of the selection process were resolved through discussion, or with an additional reviewer. The results of the search and the study inclusion process are presented in a Preferred Reporting Items for Systematic Reviews (PRISMA) and Meta‐analyses extension for scoping review flow diagram.^21^

### 2.6 Data extraction

Data were extracted from articles included in the scoping review by two or more independent reviewers using a data extraction tool developed by the reviewers. The data extraction tool was designed to capture information about the source (author, year of publication, country of study), study design, type of intervention, health topic and outcome measure of interest. Pilot extraction was undertaken with three included studies.

### 2.7 Data analysis and presentation

#### 2.7.1 Assessment of engagement

The reported presence of both CYP and parent engagement in research involving the design and/or implementation of health interventions were categorized based on five key phases of research, which were developed in consultation with experts: (1) generating a research question; (2) designing study methods; (3) collecting data; (4) interpreting results; and (5) reporting findings. An engagement score was coded for each study to represent the total reported phases of the research process with CYP involvement (0 = no phases of involvement reported to 5 = reported involvement in all phases). A similar process was undertaken to evaluate parental involvement in the included studies.

#### 2.7.2 Assessment of interventions

The Behaviour Change Wheel (BCW) was used as a framework to characterize the included interventions according to the nine intervention function types (i.e., the proposed mechanism of the intervention).^22^ The nine intervention function types are environmental restructuring, modelling, enablement, training, coercion, incentivization, persuasion and education. The BCW has been used to characterize interventions in a number of different settings.^23^, ^24^ Intervention functions types were mapped against the population that was engaged in the research process and the intervention target population. This was done to explore whether intervention approaches differed between different target and engagement populations. Two independent reviewers coded the reported intervention descriptions using the BCW. Reviewers met and came to consensus on any discrepancies in coding. If consensus could not be reached, a third reviewer was consulted.

#### 2.7.3 Assessment of barriers and enablers

Author‐reported barriers and enablers to engaging CYP in the development or implementation of health interventions were categorized using the determinants of partnership synergy, a component of the partnership synergy framework.^25^ The partnership synergy framework is a theory‐based framework designed to study and optimize the effectiveness of partnerships.^25^ The determinants of partnership synergy operationalize five determinants that contribute to high levels of synergy: (1) resources; (2) partner characteristics; (3) relationships among partners; (4) partnership characteristics; and (5) external environment. Enablers and barriers were categorized into these determinants, indicating a presence or absence, respectively. Two independent reviewers coded enablers and barriers, following similar methods utilized for the assessment of engagement and intervention functions.

### 2.8 Parent involvement

A parent partner with codesign experience was involved throughout the research process. The aim of their involvement was to inform the framing of our research question and interpretation of our results from the perspective of someone with experience in intervention design. They contributed to the creation of the research questions, screened titles, abstracts and full texts, helped with data extraction and synthesis and provided feedback on the manuscript. This review adhered to the patient involvement reporting standards outlined in the short form of the Guidance for Reporting Involvement of Patients and the Public‐2 (GRIPP‐2).^26^

## 3 RESULTS

### 3.1 Characteristics of included studies

**Figure 1:**
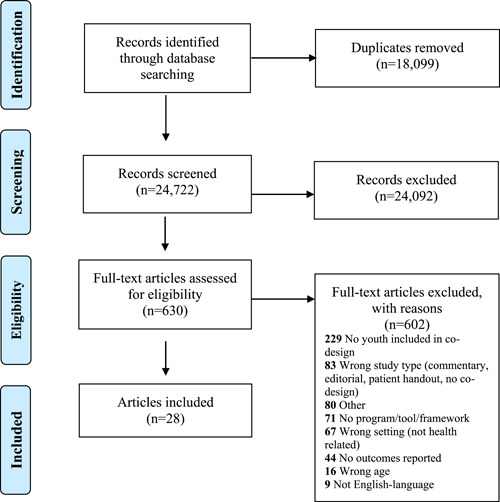
PRISMA Diagram. PRISMA, Preferred Reporting Items for Systematic Reviews and Meta‐Analyses.

Reviewers screened 42,722 titles/abstracts and reviewed 631 full‐text articles for eligibility (Figure 1). Our parent partner screened 891 abstracts and 55 full texts and extracted data from 10 articles. Twenty‐eight articles describing twenty‐four studies fulfilled the inclusion criteria. The general characteristics of the included studies are described in Table 1. Sixteen studies engaged only CYP,^27^, ^28^, ^29^, ^30^, ^31^, ^32^, ^33^, ^34^, ^35^, ^36^, ^37^, ^38^, ^39^, ^40^, ^41^, ^42^, ^43^, ^44^ while the remaining eight engaged both CYP and parents.^45^, ^46^, ^47^, ^48^, ^49^, ^50^, ^51^, ^52^, ^53^, ^54^ The research took place in community, primary and tertiary care settings and addressed a variety of health topics (e.g., sexual health, asthmas, obesity, mental health, cancer, limited mobility, visible difference). Qualitative and mixed‐methods study designs were observed most often (*n* = 22),^27^, ^28^, ^30^, ^31^, ^32^, ^33^, ^34^, ^35^, ^36^, ^38^, ^39^, ^40^, ^41^, ^42^, ^43^, ^44^, ^45^, ^46^, ^47^, ^48^, ^49^, ^50^, ^51^, ^52^, ^53^, ^54^ and all studies were guided by a diverse set of frameworks, with community‐based participatory research (*n* = 5)^29^, ^36^, ^37^, ^49^, ^51^ and participatory action research (*n* = 8)^27^, ^28^, ^32^, ^33^, ^34^, ^35^, ^39^, ^44^, ^51^ being the most common.

**Table 1:** General characteristics of included studies (*n* = 20)

Author (publication year)	Country	Setting	Health topic	Age of CYP	Study design	Frameworks used to guide study
*Studies involving CYP and parents*						
Eberhart et al. (2019)^45^	USA	Community	Asthma	≥12	Qualitative	Human‐centred design
Harrington et al. (2021)^46^	UK	Community	Diabetes—type II	12–14	Qualitative	A theoretical framework based on self‐efficacy theory and the capability, opportunity, motivation, behaviour (COM‐B) model
Loyd et al. (2017)^47^	UK	Community	Obesity	9–10	Mixed methods	Intervention mapping
Morales et al. (2018)^48^	Canada	Community	Limited mobility	12–21	Qualitative	User‐centred design
Morales‐Campos et al. (2015)^49^	USA	Community	Obesity	11–14	Qualitative	CBPR, Social cognitive theory
Pembroke et al. (2021)^50^	Ireland	Tertiary Care	Diabetes—type I	11–17	Qualitative	Social cognitive theory
Radovic et al.(2016)^51^	USA	Community	Mental health—depression	13–21	Mixed methods	CBPR, Obesity‐related behavioural intervention trials model
Ruland et al. (2006, 2007, 2008)^52^, ^53^, ^54^	Norway	Tertiary care	Cancer	9–11	Qualitative	Participatory design
*Studies involving CYP*						
Anselma et al. (2019, 2020)^27^, ^28^	Netherlands	Community	Obesity	9–12	Qualitative	Youth‐led Participatory Action Research, Intervention mapping
Bauermeister et al. (2015)^29^	USA	Community	HIV/STIs	17–24	Quantitative	CBPR, Integrated behavioural model
Braun et al. (2020)^30^	Austria	Community	Mental health—suicide	15–19	Qualitative	Suicide Awareness and Voices of Education in the United States
Chaniang et al. (2019)^31^	Thailand	Mixed methods	Mental health—suicide	12–18	Mixed methods	Action research
Dunn (2017)^32^	UK	Tertiary care	Mental health	16–22	Qualitative	Participatory research approaches
Hawkins et al. (2017)^33^	UK	Community	Mental health—substance misuse	13–19	Qualitative	Transdisciplinary Action Research
Jaume et al. (2015)^34^	UK	Community and tertiary care	General health	4–14	Qualitative	Participatory research
Lane et al. (2019)^35^	USA	Community	Obesity/diabetes—type II	11–14	Mixed methods	Youth participatory research
Livingood et al. (2017)^36^	USA	Community	Obesity	15–19	Qualitative	CBPR
Mance et al. (2010)^37^	USA	Community	Mental health	16–24	Quantitative	CBPR, Cognitive‐behavioural and stress exposure conceptual models
Patchen et al. (2020)^38^	USA	Community	Sexual health	15–21	Mixed methods	Social cognitive theory, Problem‐solving theory
Povey et al. (2020)^39^	Australia	Community	Mental health	10–18	Mixed methods	Participatory research approaches
Saini et al. (2020)^40^	Canada	Community	Acute gastrointestinal illness	11–12	Qualitative	Community engagement methods
Versnel (2011)^41^	Canada	Tertiary care	Chronic health conditions	13–15	Qualitative	Youth engagement
Watson et al. (2017)^42^; Brady et al. (2018)^43^	UK	Community	Mental health—substance misuse	16–21	Qualitative	Young People's Advisory Group
Williamson et al. (2015)^44^	UK	Community	Visible difference	12–19	Mixed methods	Participatory intervention model, participatory action research, Kent's Model of Psychosocial Distress and intervention for individuals with visible differences

Abbreviations: CBPR, community‐based participatory research; CYP, children and young people; HIV, human immunodeficiency viruses; NR, not reported; STDs, sexually transmitted diseases; STIs, sexually transmitted infections; UK, United Kingdom.

### 3.2 Presence of engagement

The CYP engagement score in research involving the design and/or implementation of health interventions ranged from 1–5. The majority of studies only reported engaging CYP during one (*n* = 13)^30^, ^32^, ^33^, ^34^, ^37^, ^38^, ^44^, ^46^, ^47^, ^48^, ^50^, ^51^, ^52^, ^53^, ^54^ or two (*n* = 6)^29^, ^31^, ^35^, ^39^, ^40^, ^41^ phases of the research process. Developing the research question and interpreting results had the lowest reported engagement of CYP, while designing methods had the highest reported engagement of CYP (Table 2). Studies that did not engage CYP in forming the research question recruited them after the research question was generated.

**Table 2:** Research phases in which CYP were engaged (*n* = 24)

Author (publication year)	Research question	Methods	Data collection	Interpretating results	Reporting results	Engagement score
*Studies involving CYP and parents*						
Eberhart et al. (2019)^45^	1	1	1	0	0	3
Harrington et al. (2021)^46^	0	0	0	1	0	1
Loyd et al. (2017)^47^	0	1	0	0	0	1
Morales et al. (2018)^48^	0	0	1	0	0	1
Morales‐Campos et al. (2015)^49^	1	1	0	1	1	4
Pembroke et al. (2021)^50^	0	0	0	1	0	1
Radovic et al. (2016)^51^	0	1	0	0	0	1
Ruland et al. (2006, 2007, 2008)^52^, ^53^, ^54^	0	1	0	0	0	1
*Studies involving CYP*						
Anselma et al. (2019, 2020)^27^, ^28^	1	0	1	1	0	3
Bauermeister et al. (2015)^29^	0	1	1	0	0	2
Braun et al. (2020)^30^	0	1	0	0	0	1
Chaniang et al. (2019)^31^	0	1	0	0	1	2
Dunn (2017)^32^	0	0	0	0	1	1
Hawkins et al. (2017)^33^	0	0	0	1	0	1
Jaume et al. (2015)^34^	0	1	0	0	0	1
Lane et al. (2019)^35^	0	1	0	0	1	2
Livingood et al. (2017)^36^	0	1	1	1	1	4
Mance et al. (2010)^37^	0	1	0	0	0	1
Patchen et al. (2020)^38^	0	1	0	0	0	1
Povey et al. (2020)^39^	0	0	1	1	0	2
Saini et al. (2020)^40^ 2020	0	1	0	0	1	2
Versnel (2011)^41^	0	1	0	0	1	2
Watson et al. (2017)^42^; Brady et al. (2018)^43^	1	1	1	1	1	5
Williamson et al. (2015)^44^	0	1	0	0	0	1

*Note*: 1 = engaged, 0 = not reported.

Abbreviation: CYP, children and young people.

Only one study, which was discussed in two articles,^42^, ^43^ reported engagement across all five phases of the research process. The youth social behaviour and network therapy study established a young people's advisory group to guide engagement, which emphasized both consultation and coproduction to facilitate opportunities for young people at each phase of the research.^42^, ^43^

Among the studies that engaged both CYP and parents, the engagement score in research involving the design and/or implementation of health interventions for parental involvement ranged from 1–3 (Table 3), with most reporting parent engagement during one phase of the research process (*n* = 6).^46^, ^47^, ^48^, ^50^, ^51^, ^52^, ^53^, ^54^ Parents were reported as being engaged in an equal or lower number of phases than CYP. For instance, Morales‐Campos et al.^49^ reported engaging CYP during the research question, methods, interpretation of results and reporting of findings, while there were only descriptions of parent engagement during two phases. Parents were always reported as being engaged concurrently with CYP, and parents were never engaged in phases in which CYP were not.

**Table 3:** Research phases in which parents were engaged (*n* = 10)

Author (publication year)	Research question	Methods	Data collection	Interpretating results	Reporting results	Engagement score
Eberhart et al. (2019)^45^	1	1	1	0	0	3
Harrington et al. (2021)^46^	0	0	0	1	0	1
Loyd et al. (2017)^47^	0	1	0	0	0	1
Morales et al. (2018)^48^	0	0	1	0	0	1
Morales‐Campos et al. (2015)^49^	0	1	0	1	0	2
Pembroke et al. (2021)^50^	0	0	0	1	0	1
Radovic et al. (2016)^51^	0	1	0	0	0	1
Ruland et al. (2006, 2007, 2008)^52^, ^53^, ^54^	0	1	0	0	0	1

*Note*: 1 = engaged, 0 = not reported.

There appeared to be no differences in the reported presence of engagement in studies engaging only CYP versus studies engaging CYP and parents.

### 3.3 Target and types of interventions

Of the 10 studies that included CYP and parents, four studies reported on interventions that targeted both CYP and parents,^31^, ^40^, ^45^, ^51^ with two of these engaging CYP and parents for more than one research phase.^45^, ^51^ The remaining six studies designed interventions for CYP only.^46^, ^47^, ^48^, ^49^, ^50^, ^53^ There was no noticeable difference between the intervention function types used when both CYP and parents were engaged compared to when only CYP were engaged (Table 4). However, the training intervention function type was only used with interventions designed to target CYP.^37^, ^42^, ^43^, ^44^ Incentivization, coercion and restriction function types were not used in any of the studies.

**Table 4:** Health intervention characteristics (*n* = 24)

Authors (publication year)	Description of the intervention	Target of the intervention	CYP engagement score	Identified BCW domains					
				Education	Persuasion	Training	Modelling	Environmental restructuring	Enablement
*Studies involving CYP and parents*									
Eberhart et al. (2019)^45^	Support system, asthma activity sheet, conversation starter pack.	CYP and parents	3					✘	
Harrington et al. (2021)^46^	An interactive lifestyle training programme.	CYP	1	✘		✘			
Loyd et al. (2017)^47^	Health education programme.	CYP	1	✘					
Morales et al. (2018)^48^	Mobility devices and environmental design solutions.	CYP	1					✘	
Morales‐Campos et al. (2015)^49^	Community physical activity programme.	CYP	4						✘
Pembroke et al. (2021)^50^	Educational videos of common questions asked during hospital visits.	CYP	1	✘					
Radovic et al. (2016)^51^	Educational websites designed to increase treatment engagement and access to an online community.	CYP and parents	1	✘	✘				
Ruland et al. (2006, 2007, 20087)^52^, ^53^, ^54^	Phone/tablet application designed to improve communication between youth and clinicians.	CYP	1					✘	
*Studies involving CYP*									
Anselma et al. (2019, 2020)^27^, ^28^	Cooking workshops, after school activities, sports events, installation of a water fountain.	CYP	3						✘
Bauermeister et al. (2015)^29^	Web application promoting HIV/STI testing.	CYP	2	✘	✘				
Braun et al. (2020)^30^	Suicide prevention videos.	CYP	1	✘					
Chaniang et al. (2019)^31^	Suicide prevention promotion and education programme.	CYP and Parents	2	✘			✘		
Dunn (2017)^32^	Transition programme for transitions from mental health services.	CYP	1	✘					
Hawkins et al. (2017)^33^	Informal peer‐led drug prevention programme.	CYP	1		✘	✘			
Jaume et al. (2015)^34^	Animated tool designed to collect health information from children.	CYP	1					✘	
Lane et al. (2019)^35^	Skill‐building and community changes to reduce sugar‐sweetened beverage intake.	CYP	2						✘
Livingood et al. (2017)^36^	A digital health promotion programme.	CYP	4	✘				✘	
Mance et al. (2010)^37^	Modified Psychotherapy for Adolescents Responding to Chronic Stress.	CYP	1			✘			
Patchen et al. (2020)^38^	A sexual health education mobile‐based video game.	CYP	1	✘					
Povey et al. (2020)^39^	An interactive care planning e‐mental health mobile app.	CYP	2						✘
Saini et al. (2020)^40^	Educational video about acute gastrointestinal illness.	CYP and parents	2	✘					
Versnel (2011)^41^	Youth wellness support networks.	CYP	2					✘	
Watson et al. (2017)^42^; Brady et al. (2018)^43^	Youth social behaviour and network therapy.	CYP	5			✘		✘	
Williamson et al. (2015)^44^	Web application that integrates cognitive behavioural therapy and social skills training.	CYP	1			✘			

Abbreviations: BCW, Behaviour Change Wheel; CYP, children and young people.

### 3.4 Reported outcomes of engagement

CYP or CYP and parent engagement outcomes were primarily captured through informal qualitative feedback from CYP, parents or observations made by the study team. No validated quantitative outcome measures related to engagement or engagement frameworks/hierarchies were used. As such, reported outcomes of engagement were entirely narrative descriptions. A summary of reported author anecdotes is presented in Table 5. Reported outcomes of engagement fell into two categories: (1) CYP or CYP and parents benefited from being engaged (i.e., gained research experience, knowledge, confidence, opportunities and changes in attitudes and behaviours) and (2) the intervention design and/or implementation were improved by CYP engagement with or without parental involvement (Table 5).

**Table 5:** Outcomes of engagement (*n* = 24)

Author (publication year)	Outcome of engagement
*Studies involving CYP and parents*	
Eberhart et al. (2019)^45^	Stakeholder involvement resulted in the development of unique and ready to implement interventions.
Harrington et al. (2021)^46^	Stakeholder involvement provided valuable feedback of session content, format and delivery.
Loyd et al. (2017)^47^	High retention rates were attributed to the involvement of children in the development of the intervention.
Morales et al. (2018)^48^	Engaging stakeholders provided a novel perspective that yielded new design solutions.
Morales‐Campos et al. (2015)^49^	Girls gained new insights into better understanding their community and the issue of increasing PA among girls their age.
Pembroke et al. (2021)^50^	Stakeholder involvement allowed for the identification of relevant intervention priorities and made involved adolescents feel empowered.
Radovic et al. (2016)^51^	Without stakeholders, investigators would have had to make major (and potentially incorrect) assumptions.
Ruland et al. (2006, 2007, 2008)^52^, ^53^, ^54^	Children contributed creative suggestions that the design team would not have thought of and improved the software.
*Studies involving CYP*	
Anselma et al. (2019, 2020)^27^, ^28^	Children's perspectives improved understanding of the issues and resulted in a more relevant intervention. Children were empowered through participation.
Bauermeister et al. (2015)^29^	Youth insight was crucial to the success of the study.
Braun et al. (2020)^30^	Involving adolescents increased the relevance of the intervention and resulted in an increased sense of well‐being.
Chaniang et al. (2019)^31^	Adolescent involvement was a key to successful programme development.
Dunn (2017)^32^	Young people gained the opportunity to think creatively about transition preparation.
Hawkins et al. (2017)^33^	Involving young people improved the acceptability, feasibility and quality of the intervention.
Jaume et al. (2015)^34^	Child involvement allowed the tool to be adjusted to children's needs.
Lane et al. (2019)^35^	Youth gained valuable knowledge through involvement.
Livingood et al. (2017)^36^	Youth contributed valuable insight into the development of the intervention.
Mance et al. (2010)^37^	Peer leaders added intervention material, making it more relevant for the target community.
Patchen et al. (2020)^38^	Youth engagement was essential to the success of the development of the intervention.
Povey et al. (2020)^39^	Young people assisted in tailoring the intervention to their preferences.
Saini et al. (2020)^40^	Partnering with youth was attributed to the success and cultural relevance of the intervention.
Versnel (2011)^41^	Youth leaders gained a sense of accomplishment and a desire to ‘do something more meaningful with my life’.
Watson et al. (2017)^42^; Brady et al. (2018)^43^	Young advisors benefited by recognizing their ability to achieve positive change, but were worried about the stigma associated with being involved in a project about mental health.
Williamson et al. (2015)^44^	Stakeholders were empowered to develop an acceptable intervention that integrates the theoretical and current evidence base regarding intervention content, with the beliefs, motivations, language, culture and practices of potential service users and HCPs

Abbreviation: CYP, children and young people.

Although most studies (*n* = 20)^27^, ^29^, ^30^, ^31^, ^32^, ^33^, ^34^, ^36^, ^37^, ^38^, ^39^, ^40^, ^44^, ^45^, ^46^, ^47^, ^48^, ^50^, ^51^, ^52^, ^53^, ^54^ noted the positive impact of CYP with or without parental involvement on health intervention design and/or implementation, limited details were provided on how they influenced the process. Furthermore, there were no apparent differences between the outcomes reported in studies with CYP and studies with CYP and parents. In one case, the word ‘stakeholders’ was used to group both CYP and parents together,^51^ resulting in a lack of emphasis on outcomes related to parents overall.

### 3.5 Reported barriers and enablers of engagement

Of the 24 included studies, most (*n* = 20)^27^, ^28^, ^30^, ^31^, ^32^, ^33^, ^34^, ^35^, ^36^, ^37^, ^38^, ^39^, ^40^, ^41^, ^42^, ^43^, ^44^, ^46^, ^47^, ^49^, ^51^, ^52^, ^53^, ^54^ reported barriers and/or enablers to engagement (Table 6). A prevalent determinant, addressed both as a barrier and as an enabler, was resources. Resources extended beyond the financial to include adequate time,^33^, ^38^, ^41^ training^52^ and involvement of key stakeholders.^27^, ^44^ Notably, involvement of community experts, healthcare experts, authority figures and policy makers was reported as an enabling factor of engagement.^27^, ^44^

**Table 6:** Barriers and enablers classified by the determinants of partnership synergy (*n* = 24)

Determinants of partnership synergy	Barriers	Enablers
Resources	Recruitment (i.e., lack of participation, selection bias).^39^, ^42^, ^49^	Involving community and healthcare experts.^27^
	Time limitations.^33^, ^38^, ^41^	Involving authority figures and policy makers.^44^
	Lack of resources.^35^	Incorporating community and regional partnerships.^28^, ^40^, ^46^
		Having adequate funding.^52^
		Providing training.^52^
		Maintaining resources and support to accommodate a variety of different backgrounds and ages.^34^
Partner characteristics	Including youth with suicide ideation during development of a suicide intervention posed health concerns.^31^	Involving CYP early on.^41^, ^42^
	Youth lacked methodological expertize.^36^	
	Youth lacked of technological skill.^30^	
	Ageing out of the study.^35^	
	Steep learning curve of subject matter.^52^, ^53^, ^54^	
	Youth and parents lacked the motivation to contribute.^28^	
Relationships among partners	Trust building.^53^	Maintaining contact throughout the research process (via email and social media in conjunction with group meetings).^42^, ^43^
	Clique formation.^52^	Researchers encouraging responsibility of youth's own care.^32^
	Conflict among stakeholders.^33^	Mentorship that values the skills and ideas of youth.^30^
	Competing priorities and goals between stakeholders.^33^	Researchers maintaining awareness of group dynamics.^51^
		Allowing engaged partners to share personal experiences.^53^
Partnership characteristics	Maintaining consistent engagement over a lengthy research process.^37^, ^49^, ^53^, ^54^	Including other lines of methodology and clinical research (engaging partners is not always sufficient to ensure a valid intervention).^54^
	Researchers balancing guidance and autonomy in terms of youth engagement.^27^, ^37^	Working in parallel with two different age groups.^52^
	Involving youth slowed down the process.^34^	Keeping flexible scheduling to accommodate engaged partners.^42^
		Using principles of colearning and shared responsibility.^37^
		Defining roles of engagement.^52^
		Iterative design, with multiple opportunities for feedback.^38^
External environment	Geographic distances between researchers and youth.^35^	
	Within a school setting, school assessment pressures and school staffing issues.^47^	

Abbreviation: CYP, children and young people.

Relationships among partners also represented a common determinant among the studies. However, the enablers and barriers under this classification were not specifically related to the parent–CYP dyad; instead, they focused on engaged partners in general, the CYP–CYP dyad or the CYP–researcher dyad. For example, one study, which engaged both CYP and parents, reported only on the CYP/parent–researcher dyad (i.e., building trust between researchers and partners and ensuring that researchers allowed engaged partners to share personal experiences).^53^

There were no discernible differences in the types of reported barriers and enablers or their frequency between articles engaging CYP and articles engaging CYP and parents.

## 4 DISCUSSION

This scoping review identified a heterogeneous body of literature and covers a wide range of health interventions. These findings are consistent with previous scoping reviews, which describe inconsistent engagement across research phases with varying levels of engagement^14^, ^15^ and inconsistent use of terminology.^16^ This review builds on previous work by providing a detailed overview of current engagement practices with CYP and CYP and parents in research involving the design and/or implementation of health interventions. Our findings show that there is little evidence to support any differences between studies that engaged CYP versus CYP and parents in the presence of engagement, the types of interventions that were designed and/or implemented and the outcomes of engagement. This review also provides novel insight into the scarcity of evidence related to how relational dynamics impact engagement and summarizes the breadth of barriers and enablers to engagement unique to the context of CYP and parent involvement in health intervention design.

CYP were rarely reported to have been engaged at every phase of the research process and parents were never reported to have been engaged in more than three phases. Engagement for both CYP and parents was mostly limited to the development and design of research methods. Although similar scoping reviews have also reported a lack of consistent stakeholder engagement, Larsson et al. found CYP to be less involved during research design, implementation and data analysis phases.^14^ Shen et al.^15^ observed a greater range of parent participation across the research spectrum including the planning, design, collection and analysis of data, and dissemination of findings; however, no study maintained parent engagement throughout the entire research process. Current engagement guidelines and frameworks promote patient involvement in all aspects of the research process, as it is considered a feature of meaningful involvement and ensures stakeholder‐oriented outcomes.^2^, ^55^, ^56^, ^57^ Yet, a disparity appears to exist between theory and the reported practice for sustaining CYP and parent engagement throughout the research process. Whether this disparity is the result of underreporting or lack of adherence to engagement guidelines is unclear. It is possible that increasing the use of reporting guidelines for engaging patients in research could improve reporting of engagement practices and could encourage greater engagement and collaboration throughout the entirety of the research process.

Researchers struggle with determining how to authentically engage CYP as coinvestigators, and adding parents to the process can result in an additional layer of complexity.^58^ While some advancements can be seen with regard to effective ways to engage CYP, there is growing support to also demonstrate the added value of incorporating parents with CYP in the design and implementation of programmes or interventions related to CYP health (e.g., parents can challenge assumptions underlying research priorities and provide first‐hand perspectives).^59^, ^60^, ^61^, ^62^, ^63^ It is recognized that engagement should extend beyond the patient to their family^64^ and that relational dynamics inform engagement.^65^ However, in practice, limited strides have been made toward the greater inclusion of parents within the field of codesigning health intervention for CYP.^15^ Further, there remains a scarcity of literature concerning dyadic activation and engagement of patients with their caregivers.^66^ Future work should examine how to synergistically bring CYP and parents together as important stakeholders within the research team.

All of the included studies reported on the benefits of engagement for CYP or CYP and parents and/or the interventions. Successful experiences were most often enabled by the presence of sufficient resources (e.g., funds, training, involvement of relevant community members)^27^, ^44^, ^52^ and supporting relationships among partners (e.g., mentorship, awareness of group dynamics, maintaining contact throughout the research process).^30^, ^42^, ^43^, ^51^ Research environment, expectations, support and value have been identified by patients and their families as essential factors to ensuring meaningful engagements as partners on research teams.^67^ Future engagement research should specifically plan how to fund and support engagement opportunities.

In line with the findings from Flynn et al.,^16^ this review encountered substantial variation in reporting standards among the included studies, which made comparisons across papers difficult. The lack of standardization of key terms describing ‘engagement’ in research made it challenging to clearly distinguish studies that fulfilled the inclusion criteria. The inconsistent use of language is particularly pertinent to scoping and systematic reviews since diverse terminology used to define engagement can make it more difficult to find existing literature and potentially problematic when determining the level of stakeholder engagement.^15^ Implementing standards in nomenclature would help in clarifying issues arising from inconsistent use of terminology and has been identified as an important next step in other scoping reviews of integrated knowledge user engagement.^15^, ^68^ In addition, adherence to the Consolidated Standards of Reporting Trials statement^69^ for clinical trials, the PRISMA statement^70^ for systematic reviews and meta‐analyses or other applicable reporting guidelines is strongly encouraged to improve the transparency of publication in health intervention research.

### 4.1 Parent engagement

Our parent partner provided valuable insight into our findings, which helped contextualize and inform the presentation of our results. They held an integral role throughout this review, being involved in each stage of the process. While our parent partner was an essential aspect of this review, there were several challenges. In line with our findings, for those with little to no research experience, there can be a steep learning curve in understanding research methodology. Additionally, scientific jargon can, at times, make communication less efficient, as more time has to be spent clarifying concepts.

### 4.2 Limitations

This study had several limitations. The search was limited to the English language only. In addition, given the diverse use of terminology related to CYP engagement, it is possible that we missed some relevant literature. However, we kept our search strategy broad with the intent of capturing hard to reach reports. It is possible that some relevant literature may have been omitted during screening due to CYP or parents not being mentioned in the abstract. However, our implementation of broad inclusion criteria ensured that a wide range of literature was captured and included in our review. We appreciate that the absence of reporting does not necessarily indicate the absence of engagement. Adherence to reporting standards such as the GRIPP‐2 would strengthen our synthesis work related to this topic. The use of engagement hierarchies, such as Hart's Ladder of Youth Participation^20^ or Shier's Pathways to Participation Model,^19^ could potentially be valuable tools in defining levels of engagement for future researchers. Although our review team did include a parent and researchers and healthcare providers who work with children and young adults, a CYP was not directly involved in this review. Also, while this review pertains to more systems‐level content, our review team did not include any decision‐makers.

## 5 CONCLUSION

Our findings suggest that engagement of CYP, with or without parental involvement, in designing and/or implementing health interventions is limited. While CYP have been engaged in decisions regarding intervention components, they are seldom engaged throughout the research process, which may hinder meaningful involvement and the inclusion of patient‐reported outcomes. Further, we do not yet know if parental engagement alongside CYP may alter the nature of CYP engagement as this was not addressed in the study reports. While more engagement can create barriers, researchers should consider how the perspectives of CYP and their parents can strengthen the research process beyond the design of research methods, as well as the impact of dyadic engagement. This scoping review provides foundational knowledge on the enablers and barriers of CYP engagement, both with and without parents, for health intervention research. Our findings serve as a valuable tool for future intervention development and offer avenues for further exploration of appropriate engagement practices of both CYP and parents during health intervention design and implementation.

## AUTHOR CONTRIBUTIONS

Daniel Crowther contributed to article screening, data extraction and data analysis, and drafted the manuscript. Holly McCulloch, Helen Wong and Catie Johnson contributed to article screening and data extraction, and reviewed and edited the manuscript. Dr. Jill Chorney and Dr. Krista Ritchie developed the research questions, contributed to data analysis and reviewed and edited the manuscript. Rebecca Mackay, Dr. Logan Lawrence and Dr. Andrea Bishop developed the research questions, contributed to article screening, data extraction and data analysis, and reviewed and edited the manuscript. Melissa Helwig developed the search strategy, contributed to article screening and reviewed and edited the manuscript. Dr. Janet Curran developed the research questions, contributed to article screening and data analysis and reviewed and edited the manuscript.

## CONFLICT OF INTEREST

The authors declare no conflict of interest.

## Supporting information

Supporting information.

Supporting information.

Supporting information.

## Data Availability

The data that support the findings of this study are available from the corresponding author upon reasonable request.

## References

[1] Canadian Institutes of Health Research . Strategy for patient‐oriented research—CIHR. April 1, 2019. Accessed March 19, 2019. http://www.cihr-irsc.gc.ca/e/41204.html

[2] Government of Canada CI of HR . Strategy for patient‐oriented research—patient engagement framework—CIHR. July 2, 2014. Accessed July 8, 2020. https://cihr-irsc.gc.ca/e/48413.html#a4

[3] National Institute for Health Research . NIHR annual report 2015/16: National Institute for Health Research. Accessed March 18, 2019. https://www.nihr.ac.uk/about-us/documents/NIHR-Annual-Report-2015-16.pdf

[4] Sheridan S , Schrandt S , Forsythe L , Hilliard TS , Paez KA , Advisory Panel on Patient Engagement (2013 Inaugural Panel) . The PCORI engagement rubric: promising practices for partnering in research. Ann Fam Med. 2017;15(2):165‐170. 10.1370/afm.2042 28289118 PMC5348236

[5] National Institute of Health Research . INVOLVE | INVOLVE supporting public involvement in NHS, public health and social care research. Accessed March 18, 2019. https://www.invo.org.uk/

[6] Patient‐Centered Outcomes Research Institute (PCORI) . Examining How COVID‐19 Impacts Children in Communities of Color. Accessed July 8, 2020. https://www.pcori.org/

[7] Esmail L , Moore E , Rein A . Evaluating patient and stakeholder engagement in research: moving from theory to practice. J Comp Eff Res. 2015;4(2):133‐145. 10.2217/cer.14.79 25825842

[8] Manafo E , Petermann L , Mason‐Lai P , Vandall‐Walker V . Patient engagement in Canada: a scoping review of the ‘how’ and ‘what’ of patient engagement in health research. Health Res Policy Syst. 2018;16(1):5. 10.1186/s12961-018-0282-4 29415734 PMC5804082

[9] United Nations . Youth. Accessed January 28, 2022. https://www.un.org/en/global-issues/youth

[10] Nygren JM , Lindberg S , Wärnestål P , Svedberg P . Involving children with cancer in health promotive research: a case study describing why, what, and how. JMIR Res Protoc. 2017;6(2):e19. 10.2196/resprot.7094 28174150 PMC5320392

[11] Clarke SA . “Child's rights perspective”: the “right” of children and young people to participate in health care research. Issues Compr Pediatr Nurs. 2015;38(3):161‐180. 10.3109/01460862.2015.1042171 26331448

[12] Coyne I . Accessing children as research participants: examining the role of gatekeepers. Child Care Health Dev. 2010;36(4):452‐454. 10.1111/j.1365-2214.2009.01012.x 20642564

[13] Daly W . “Adding their flavour to the mix”: involving children and young people in care in research design. Aust Soc Work. 2009;62(4):460‐475. 10.1080/03124070903265732

[14] Larsson I , Staland‐Nyman C , Svedberg P , Nygren JM , Carlsson IM . Children and young people's participation in developing interventions in health and well‐being: a scoping review. BMC Health Serv Res. 2018;18(1):507. 10.1186/s12913-018-3219-2 29954392 PMC6027768

[15] Shen S , Doyle‐Thomas KAR , Beesley L , et al. How and why should we engage parents as co‐researchers in health research? A scoping review of current practices. Health Expect Int J Public Particip Health Care Health Policy. 2016;20:543‐554. 10.1111/hex.12490 PMC5513005 27516003

[16] Flynn R , Walton S , Scott SD . Engaging children and families in pediatric health research: a scoping review. Res Involv Engagem. 2019;5(1):32. 10.1186/s40900-019-0168-9 31700676 PMC6827239

[17] Aromataris E , Munn Z , eds. JBI Manual for Evidence Synthesis. JBI; 2020. 10.46658/JBIMES-20-01

[18] Government of Canada CI of HR . Guide to knowledge translation planning at CIHR: integrated and end‐of‐grant approaches—CIHR. December 6, 2012. Accessed July 8, 2020. https://cihr-irsc.gc.ca/e/45321.html

[19] Shier H . Pathways to participation: openings, opportunities and obligations. Child Soc. 2001;15(2):107‐117. 10.1002/chi.617

[20] Hart RA . Children's Participation: From Tokenism to Citizenship. UNICEF, International Child Development Centre; 1992.

[21] Tricco AC , Lillie E , Zarin W , et al. PRISMA Extension for Scoping Reviews (PRISMA‐ScR): checklist and explanation. Ann Intern Med. 2018;169(7):467‐473. 10.7326/M18-0850 30178033

[22] Michie S , Atkins L , West R . The Behaviour Change Wheel (Behavior Change Wheel)—A Guide to Designing Interventions. Silverback Publishing; 2014.

[23] Presseau J , Ivers NM , Newham JJ , Knittle K , Danko KJ , Grimshaw JM . Using a behaviour change techniques taxonomy to identify active ingredients within trials of implementation interventions for diabetes care. Implement Sci. 2015;10:55. 10.1186/s13012-015-0248-7 25900104 PMC4438476

[24] Curran JA , Gallant AJ , Zemek R , et al. Discharge communication practices in pediatric emergency care: a systematic review and narrative synthesis. Syst Rev. 2019;8(1):83. 10.1186/s13643-019-0995-7 30944038 PMC6446263

[25] Lasker RD , Weiss ES , Miller R . Partnership synergy: a practical framework for studying and strengthening the collaborative advantage. Milbank Q. 2001;79(2):179‐205. 10.1111/1468-0009.00203 11439464 PMC2751192

[26] Staniszewska S , Brett J , Simera I , et al. GRIPP2 reporting checklists: tools to improve reporting of patient and public involvement in research. Res Involv Engagem. 2017;3(1):13. 10.1186/s40900-017-0062-2 29062538 PMC5611595

[27] Anselma M , Altenburg TM , Emke H , et al. Co‐designing obesity prevention interventions together with children: intervention mapping meets youth‐led participatory action research. Int J Behav Nutr Phys Act. 2019;16(1):1‐15. 10.1186/s12966-019-0891-5 31831006 PMC6909512

[28] Anselma M , Chinapaw MJM , Altenburg TM . “Not only adults can make good decisions, we as children can do that as well” evaluating the process of the youth‐led participatory action research ‘kids in action’. Int J Environ Res Public Health. 2020;17:625. 10.3390/ijerph17020625 31963706 PMC7014142

[29] Bauermeister JA , Pingel ES , Jadwin‐Cakmak L , et al. Acceptability and preliminary efficacy of a tailored online HIV/STI testing intervention for young men who have sex with men: the get connected! program. AIDS Behav. 2015;19(10):1860‐1874. 10.1007/s10461-015-1009-y 25638038 PMC4522230

[30] Braun M , Till B , Pirkis J , Niederkrotenthaler T . Suicide prevention videos developed by and for adolescents: a qualitative study. Crisis. 2020;42:114‐120. 10.1027/0227-5910/a000696 32431196

[31] Chaniang S , Fongkaew W , Stone TE , Sethabouppha H , Lirtmunlikaporn S . Development and evaluation of a Suicide Prevention Program for secondary school students. Pac Rim Int J Nurs Res. 2019;23(3):201‐213.

[32] Dunn V . Young people, mental health practitioners and researchers co‐produce a transition preparation programme to improve outcomes and experience for young people leaving Child and Adolescent Mental Health Services (CAMHS). BMC Health Serv Res. 2017;17:293. 10.1186/s12913-017-2221-4 28424061 PMC5397792

[33] Hawkins J , Madden K , Fletcher A , et al. Development of a framework for the co‐production and prototyping of public health interventions. BMC Public Health. 2017;17(1):689. 10.1186/s12889-017-4695-8 28870192 PMC5583990

[34] Jaume N , Abbiss M , Wray J , Ashworth J , Brown KL , Cairns J . CHILDSPLA: a collaboration between children and researchers to design and animate health states. Child Care Health Dev. 2015;41(6):1140‐1151. 10.1111/cch.12280 26227090

[35] Lane HG , Porter KJ , Hecht E , Harris P , Zoellner JM . A participatory process to engage Appalachian youth in reducing sugar‐sweetened beverage consumption. Health Promot Pract. 2019;20(2):258‐268. 10.1177/1524839918762123 29577771 PMC6119513

[36] Livingood WC , Monticalvo D , Bernhardt JM , et al. Engaging adolescents through participatory and qualitative research methods to develop a digital communication intervention to reduce adolescent obesity. Health Educ Behav. 2017;44(4):570‐580. 10.1177/1090198116677216 27811164

[37] Mance GA , Mendelson T , Iii BB , Jones J , Tandon D . Utilizing community‐based participatory research to adapt a mental health intervention for African American emerging adults. Prog Community Health Partnersh. 2010;4(2):131‐140. 10.1353/cpr.0.0112 20543488

[38] Patchen L , Ellis L , Ma TX , et al. Engaging African American youth in the development of a serious mobile game for sexual health education: mixed methods study. JMIR Serious Games. 2020;8(1):e16254. 10.2196/16254 32012041 PMC7055799

[39] Povey J , Sweet M , Nagel T , et al. Drafting the Aboriginal and Islander Mental Health Initiative for Youth (AIMhi‐Y) app: results of a formative mixed methods study. Internet Interv. 2020;21:100318. 10.1016/j.invent.2020.100318 32477884 PMC7251767

[40] Saini M , Roche S , Papadopoulos A , et al. Promoting inuit health through a participatory whiteboard video. Can J Public Health. 2020;111(1):50‐59. 10.17269/s41997-019-00189-1 31025298 PMC7046868

[41] Versnel J . You're in charge: engaging youth in designing and delivering an early preparation self‐management program. Occup Ther Ott. 2011;13(5):28‐29.

[42] Watson J , Toner P , Day E , et al. Youth social behaviour and network therapy (Y‐SBNT): adaptation of a family and social network intervention for young people who misuse alcohol and drugs—a randomised controlled feasibility trial. Health Technol Assess. 2017;21(15):1‐260. 10.3310/hta21150 PMC5402218 28399988

[43] Brady LM , Templeton L , Toner P , et al. Involving young people in drug and alcohol research. Drugs Alcohol Today. 2018;18(1):28‐38. 10.1108/DAT-08-2017-0039

[44] Williamson H , Griffiths C , Harcourt D . Developing young person's face IT: online psychosocial support for adolescents struggling with conditions or injuries affecting their appearance. Health Psychol Open. 2015;2(2):205510291561909. 10.1177/2055102915619092 PMC5193303 28070380

[45] Eberhart A , Slogeris B , Sadreameli SC , Jassal MS . Using a human‐centered design approach for collaborative decision‐making in pediatric asthma care. Public Health. 2019;170:129‐132. 10.1016/j.puhe.2019.03.004 31035123

[46] Harrington DM , Brady EM , Weihrauch‐Bluher S , et al. Development of an interactive lifestyle programme for adolescents at risk of developing type 2 diabetes: PRE‐STARt. Children. 2021;8(2):69. 10.3390/children8020069 33494347 PMC7912284

[47] Lloyd J , McHugh C , Minton J , Eke H , Wyatt K . The impact of active stakeholder involvement on recruitment, retention and engagement of schools, children and their families in the cluster randomised controlled trial of the healthy lifestyles programme (HeLP): a school‐based intervention to prevent obesity. Trials. 2017;18:378. 10.1186/s13063-017-2122-1 28807006 PMC5557526

[48] Morales E , Lindsay S , Edwards G , et al. Addressing challenges for youths with mobility devices in winter conditions. Disabil Rehabil. 2018;40(1):21‐27. 10.1080/09638288.2016.1239768 27927034

[49] Morales‐Campos DY , Parra‐Medina D , Esparza LA . Picture this!: using participatory photo mapping with Hispanic girls. Fam Community Health. 2015;38(1):44‐54. 10.1097/FCH.0000000000000059 25423243 PMC4244704

[50] Pembroke S , Roche EF , Sleath B , et al. Developing a video intervention to improve youth question‐asking and provider education during paediatric diabetes clinic encounters: the promoting adolescents communication and engagement study. Patient Educ Couns. 2021;104(9):2170‐2176. 10.1016/j.pec.2021.02.021 33640232

[51] Radovic A , DeMand AL , Gmelin T , Stein BD , Miller E . SOVA: design of a stakeholder informed social media website for depressed adolescents and their parents. J Technol Hum Serv. 2017;35(3):169‐182. 10.1080/15228835.2017.1347552 29743822 PMC5937703

[52] Ruland CM , Slaughter L , Starren J , Vatne TM . Children as design partners in the development of a support system for children with cancer. Stud Health Technol Inform. 2006;122:80‐85. 17102222

[53] Ruland CM , Slaughter L , Starren J , Vatne TM , Moe EY . Children's contributions to designing a communication tool for children with cancer. Stud Health Technol Inform. 2007;129(Pt 2):977‐982. 17911861

[54] Ruland CM , Starren J , Vatne TM . Participatory design with children in the development of a support system for patient‐centered care in pediatric oncology. J Biomed Inform. 2008;41(4):624‐635. 10.1016/j.jbi.2007.10.004 18082468

[55] Kirwan JR , de Wit M , Frank L , et al. Emerging guidelines for patient engagement in research. 2017;20(3):481‐486. 10.1016/j.jval.2016.10.003 28292494

[56] Armstrong MJ , Rueda JD , Gronseth GS , Mullins CD . Framework for enhancing clinical practice guidelines through continuous patient engagement. Health Expect. 2017;20(1):3‐10. 10.1111/hex.12467 27115476 PMC5217879

[57] Nápoles AM . Methods for translating evidence‐based behavioral interventions for health‐disparity communities. Prev Chronic Dis. 2013;10:E193. 10.5888/pcd10.130133 24262025 PMC3839588

[58] Yip JC , Clegg T , Ahn J , et al. The evolution of engagements and social bonds during child‐parent co‐design. *Proceedings of the 2016 CHI Conference on Human Factors in Computing Systems*. CHI '16. ACM; 2016. pp. 3607‐3619. 10.1145/2858036.2858380

[59] Haine‐Schlagel R , Walsh NE . A review of parent participation engagement in child and family mental health treatment. Clin Child Fam Psychol Rev. 2015;18(2):133‐150. 10.1007/s10567-015-0182-x 25726421 PMC4433419

[60] Luff D , Allair B , Litterer K , et al. Parent and teen engagement in pediatric health services research training. Acad Pediatr. 2016;16(5):496‐498. 10.1016/j.acap.2016.02.004 27095673 PMC4931977

[61] Amirav I , Vandall‐Walker V , Rasiah J , Saunders L . Patient and researcher engagement in health research: a parent's perspective. Pediatrics. 2017;140(3):e20164127. 10.1542/peds.2016-4127 28851740

[62] Jurkowski JM , Green Mills LL , Lawson HA , Bovenzi MC , Quartimon R , Davison KK . Engaging low‐income parents in childhood obesity prevention from start to finish: a case study. J Community Health. 2013;38(1):1‐11. 10.1007/s10900-012-9573-9 22714670 PMC3547242

[63] Liberati A . Need to realign patient‐oriented and commercial and academic research. Lancet Lond. 2011;378(9805):1777‐1778. 10.1016/S0140-6736(11)61772-8 22098852

[64] Carman KL , Dardess P , Maurer M , et al. Patient and family engagement: a framework for understanding the elements and developing interventions and policies. Health Aff. 2013;32(2):223‐231. 10.1377/hlthaff.2012.1133 23381514

[65] Donato S , Bertoni A . A relational perspective on patient engagement: suggestions from couple‐based research and intervention. In: Management Association, Information Resources, ed. Health Care Delivery and Clinical Science: Concepts, Methodologies, Tools, and Applications. IGI Global; 2017:1‐24. 10.4018/978-1-5225-3926-1.ch001

[66] Saita E , Acquati C , Molgora S . Promoting patient and caregiver engagement to care in cancer. Front Psychol. 2016;7:1660. 10.3389/fpsyg.2016.01660 27826279 PMC5079095

[67] Black A , Strain K , Wallsworth C , et al. What constitutes meaningful engagement for patients and families as partners on research teams? J Health Serv Res Policy. 2018;23(3):158‐167. 10.1177/1355819618762960 29504424 PMC6041763

[68] Lawrence LM , Bishop A , Curran J . Integrated knowledge translation with public health policy makers: a scoping review. Healthc Policy. 2019;14(3):55‐77. 10.12927/hcpol.2019.25792 31017866 PMC7008688

[69] Moher D , Hopewell S , Schulz KF , et al. CONSORT 2010 explanation and elaboration: updated guidelines for reporting parallel group randomised trials. BMJ. 2010;340:c869. 10.1136/bmj.c869 20332511 PMC2844943

[70] Liberati A , Altman DG , Tetzlaff J , et al. The PRISMA statement for reporting systematic reviews and meta‐analyses of studies that evaluate healthcare interventions: explanation and elaboration. BMJ. 2009;339:b2700. 10.1136/bmj.b2700 19622552 PMC2714672

