# Supplementary material for: Children, young people and parent engagement in health intervention design and implementation: A scoping review
Source: Health Expect. 2022 Nov 8;26(1):1–15. doi: 10.1111/hex.13572 (PMC9854306; doi:10.1111/hex.13572)
Supplement: Supplementary file 1 — Supporting information. [file HEX-26--s002.docx]

**Appendix Table 1: Embase Search**

| **No.** | **Query** |
| --- | --- |
| 1 | participatory research'/exp |
| 2 | 'patient participation'/exp AND 'research'/exp |
| 3 | 'patient participation'/exp |
| 4 | design*:ti,ab OR research:ti,ab OR approach*:ti,ab OR method*:ti,ab OR process*:ti,ab OR framework*:ti,ab OR tool*:ti,ab OR 'co design*':ti,ab OR codesign*:ti,ab OR 'co research*':ti,ab |
| 5 | #3 AND 4 |
| 6 | ((participatory OR participative OR participation OR cooperative* OR 'co operative*' OR 'user led' OR advocate OR advocacy OR activism OR leadership OR peer OR peers OR mentor OR client OR stakeholder OR 'user involvement') NEAR/2 (design* OR research OR approach* OR method* OR process* OR framework* OR tool* OR 'co design*' OR codesign* OR 'co research*' OR engagement OR involvement)):ti,ab |
| 7 | #1 OR #2 OR #5 OR #6 |
| 8 | 'parent'/exp |
| 9 | 'caregiver'/de |
| 10 | 'legal guardian'/de |
| 11 | parent*:ti,ab OR father*:ti,ab OR mother*:ti,ab OR guardian*:ti,ab OR caregiver*:ti,ab |
| 12 | #8 OR #9 OR #10 OR #11 |
| 13 | 'child'/exp |
| 14 | 'adolescent'/exp |
| 15 | 'child health care'/de |
| 16 | child:ti,ab OR child*:ti,ab OR schoolchild:ti,ab OR schoolchild*:ti,ab OR 'school child':ti,ab OR 'school child*':ti,ab OR kid:ti,ab OR kids:ti,ab OR toddler*:ti,ab OR adolescent:ti,ab OR adoles*:ti,ab OR teen*:ti,ab OR boy*:ti,ab OR girl*:ti,ab OR minors:ti,ab OR minor:ti,ab OR 'young adult':ti,ab OR tween:ti,ab OR underag*:ti,ab OR juvenil*:ti,ab OR youth*:ti,ab OR kindergar*:ti,ab OR puberty:ti,ab OR puber*:ti,ab OR pubescen*:ti,ab OR prepubescen*:ti,ab OR prepubert*:ti,ab OR pediatric*:ti,ab OR paediatric*:ti,ab OR schools:ti,ab OR 'nursery school*':ti,ab OR preschool*:ti,ab OR 'pre school':ti,ab OR 'primary school*':ti,ab OR 'secondary school*':ti,ab OR 'elementary school*':ti,ab OR 'high school*':ti,ab OR highschool*:ti,ab OR 'school age':ti,ab OR schoolage*:ti,ab |
| 17 | #13 OR #14 OR #15 OR #16 |
| 18 | #12 OR #17 |
| 19 | #7 AND #18 |

**Appendix Table 2: Medline Search**

| **No.** | **Query** |
| --- | --- |
| 1 | ((Participatory or Participative or Participation or Cooperative* or Cooperative* or user-led or advocate or activism or leadership or peer or peers or mentor or client or stakeholder or "user involvement") adj2 (design* or research or approach* or method* or process* or framework* or tool* or Co-design* or Codesign* or Co-research* or engagement or involvement)).ti,ab. |
| 2 | exp Community-Based Participatory Research/ |
| 3 | patient participation/ |
| 4 | 1 or 2 or 3 |
| 5 | exp Parents/ |
| 6 | Caregivers/ |
| 7 | Legal Guardians/ |
| 8 | (parent* or father* or mother* or guardian* or caregiver*).ti,ab. |
| 9 | 5 or 6 or 7 or 8 |
| 10 | (child or child* or schoolchild* or schoolchild or school child or school child* or kid or kids or toddler* or adolescent or adoles* or teen* or boy* or girl* or minor or minors or "young adult" or tween or underag* or juvenil* or youth* or kindergar* or puberty or puber* or pubescen* or prepubescen* or prepuberty* or pediatric* or paediatric* or schools or nursery school* or preschool* or pre school* or primary school* or secondary school* or elementary school* or elementary school or high school* or highschool* or school age or schoolage or school age* or schoolage*).ti,ab. |
| 11 | exp Child/ |
| 12 | exp Adolescent/ |
| 13 | Child Health Services/ or Adolescent Health Services/ |
| 14 | 10 or 11 or 12 or 13 |
| 15 | 9 or 14 |
| 16 | 4 and 15 |
| 17 | (design* or research or approach* or method* or process* or framework* or tool* or Co-design* or Codesign* or Co-research*).ti,ab. |
| 18 | exp Patient Participation/ |
| 19 | 17 and 18 |
| 20 | exp Research/ |
| 21 | 18 and 20 |
| 22 | 1 or 2 or 19 or 21 |
| 23 | 15 and 22 |

**Appendix Table 3: CINAHL Search**

| **No.** | **Query** | **Limiters/Expanders** | **Last Run Via** |
| --- | --- | --- | --- |
| S1 | (MH "Consumer Participation") | Expanders - Apply related words | Interface - EBSCOhost Research Databases |
|  |  | Search modes - Boolean/Phrase | Search Screen - Advanced Search |
|  |  |  | Database - CINAHL with Full Text |
| S2 | (MH "Research+") | Expanders - Apply related words | Interface - EBSCOhost Research Databases |
|  |  | Search modes - Boolean/Phrase | Search Screen - Advanced Search |
|  |  |  | Database - CINAHL with Full Text |
| S3 | S1 AND S2 | Expanders - Apply related words | Interface - EBSCOhost Research Databases |
|  |  | Search modes - Boolean/Phrase | Search Screen - Advanced Search |
|  |  |  | Database - CINAHL with Full Text |
| S4 | TI ( design* or research or approach* or method* or process* or framework* or tool* or Co-design* or Codesign* or Co-research* ) OR AB ( design* or research or approach* or method* or process* or framework* or tool* or Co-design* or Codesign* or Co-research* ) | Expanders - Apply related words | Interface - EBSCOhost Research Databases |
|  |  | Search modes - Boolean/Phrase | Search Screen - Advanced Search |
|  |  |  | Database - CINAHL with Full Text |
| S5 | S1 AND S4 | Expanders - Apply related words | Interface - EBSCOhost Research Databases |
|  |  | Search modes - Boolean/Phrase | Search Screen - Advanced Search |
|  |  |  | Database - CINAHL with Full Text |
| S6 | TI ( (Participatory or Participative or Participation or Cooperative* or Cooperative* or user-led or advocate or activism or leadership or peer or peers or mentor or client or stakeholder or "user involvement") N2 (design* or research or approach* or method* or process* or framework* or tool* or Co-design* or Codesign* or Co-research* or engagement or involvement) ) OR AB ( (Participatory or Participative or Participation or Cooperative* or Cooperative* or user-led or advocate or activism or leadership or peer or peers or mentor or client or stakeholder or "user involvement") N2 (design* or research or approach* or method* or process* or framework* or tool* or Co-design* or Codesign* or Co-research* or engagement or involvement) ) | Expanders - Apply related words | Interface - EBSCOhost Research Databases |
|  |  | Search modes - Boolean/Phrase | Search Screen - Advanced Search |
|  |  |  | Database - CINAHL with Full Text |
| S7 | TI community based participatory research OR AB community based participatory research | Expanders - Apply related words | Interface - EBSCOhost Research Databases |
|  |  | Search modes - Boolean/Phrase | Search Screen - Advanced Search |
|  |  |  | Database - CINAHL with Full Text |
| S8 | S3 OR S5 OR S6 OR S7 | Expanders - Apply related words | Interface - EBSCOhost Research Databases |
|  |  | Search modes - Boolean/Phrase | Search Screen - Advanced Search |
|  |  |  | Database - CINAHL with Full Text |
| S9 | (MH "Parents+") | Expanders - Apply related words | Interface - EBSCOhost Research Databases |
|  |  | Search modes - Boolean/Phrase | Search Screen - Advanced Search |
|  |  |  | Database - CINAHL with Full Text |
| S10 | (MH "Caregivers") | Expanders - Apply related words | Interface - EBSCOhost Research Databases |
|  |  | Search modes - Boolean/Phrase | Search Screen - Advanced Search |
|  |  |  | Database - CINAHL with Full Text |
| S11 | (MH "Guardianship, Legal+") | Expanders - Apply related words | Interface - EBSCOhost Research Databases |
|  |  | Search modes - Boolean/Phrase | Search Screen - Advanced Search |
|  |  |  | Database - CINAHL with Full Text |
| S12 | TI ( parent* or father* or mother* or guardian* or caregiver* ) OR AB ( parent* or father* or mother* or guardian* or caregiver* ) | Expanders - Apply related words | Interface - EBSCOhost Research Databases |
|  |  | Search modes - Boolean/Phrase | Search Screen - Advanced Search |
|  |  |  | Database - CINAHL with Full Text |
| S13 | S9 OR S10 OR S11 OR S12 | Expanders - Apply related words | Interface - EBSCOhost Research Databases |
|  |  | Search modes - Boolean/Phrase | Search Screen - Advanced Search |
|  |  |  | Database - CINAHL with Full Text |
| S14 | (MH "Child+") | Expanders - Apply related words | Interface - EBSCOhost Research Databases |
|  |  | Search modes - Boolean/Phrase | Search Screen - Advanced Search |
|  |  |  | Database - CINAHL with Full Text |
| S15 | (MH "Adolescence+") | Expanders - Apply related words | Interface - EBSCOhost Research Databases |
|  |  | Search modes - Boolean/Phrase | Search Screen - Advanced Search |
|  |  |  | Database - CINAHL with Full Text |
| S16 | (MH "Adolescent Health Services") | Expanders - Apply related words | Interface - EBSCOhost Research Databases |
|  |  | Search modes - Boolean/Phrase | Search Screen - Advanced Search |
|  |  |  | Database - CINAHL with Full Text |
| S17 | (MH "Child Health Services") | Expanders - Apply related words | Interface - EBSCOhost Research Databases |
|  |  | Search modes - Boolean/Phrase | Search Screen - Advanced Search |
|  |  |  | Database - CINAHL with Full Text |
| S18 | TI ( child or child* or schoolchild* or schoolchild or school child or school child* or kid or kids or toddler* or adolescent or adoles* or teen* or boy* or girl* or minor or minors or "young adult" or tween or underag* or juvenil* or youth* or kindergar* or puberty or puber* or pubescen* or prepubescen* or prepuberty* or pediatric* or paediatric* or schools or nursery school* or preschool* or pre school* or primary school* or secondary school* or elementary school* or elementary school or high school* or highschool* or school age or schoolage or school age* or schoolage* ) OR AB ( child or child* or schoolchild* or schoolchild or school child or school child* or kid or kids or toddler* or adolescent or adoles* or teen* or boy* or girl* or minor or minors or "young adult" or tween or underag* or juvenil* or youth* or kindergar* or puberty or puber* or pubescen* or prepubescen* or prepuberty* or pediatric* or paediatric* or schools or nursery school* or preschool* or pre school* or primary school* or secondary school* or elementary school* or elementary school or high school* or highschool* or school age or schoolage or school age* or schoolage* ) | Expanders - Apply related words | Interface - EBSCOhost Research Databases |
|  |  | Search modes - Boolean/Phrase | Search Screen - Advanced Search |
|  |  |  | Database - CINAHL with Full Text |
| S19 | S14 OR S15 OR S16 OR S17 OR S18 | Expanders - Apply related words | Interface - EBSCOhost Research Databases |
|  |  | Search modes - Boolean/Phrase | Search Screen - Advanced Search |
|  |  |  | Database - CINAHL with Full Text |
| S20 | S13 OR S19 | Expanders - Apply related words | Interface - EBSCOhost Research Databases |
|  |  | Search modes - Boolean/Phrase | Search Screen - Advanced Search |
|  |  |  | Database - CINAHL with Full Text |
| S21 | S8 AND S20 | Expanders - Apply related words | Interface - EBSCOhost Research Databases |
|  |  | Search modes - Boolean/Phrase | Search Screen - Advanced Search |
|  |  |  | Database - CINAHL with Full Text |
