# Supplementary material for: Children, young people and parent engagement in health intervention design and implementation: A scoping review
Source: Health Expect. 2022 Nov 8;26(1):1–15. doi: 10.1111/hex.13572 (PMC9854306; doi:10.1111/hex.13572)
Supplement: Supplementary file 2 — Supporting information. [file HEX-26--s003.docx]

| **Section and topic** | **Item** | **Reported on page No** |
| --- | --- | --- |
| 1: Aim | Report the aim of PPI in the study | Page 6 |
| 2: Methods | Provide a clear description of the methods used for PPI in the study | Page 6 |
| 3: Study results | Outcomes—Report the results of PPI in the study, including both positive and negative outcomes | Page 6 |
| 4: Discussion and conclusions | Outcomes—Comment on the extent to which PPI influenced the study overall. Describe positive and negative effects | Page 22-23 |
| 5: Reflections/critical perspective | Comment critically on the study, reflecting on the things that went well and those that did not, so others can learn from this experience | Page 22-23 |

Taken from: Staniszewska, S., Brett, J., Simera, I. *et al.* GRIPP2 reporting checklists: tools to improve reporting of patient and public involvement in research. *Res Involv Engagem* **3,**13 (2017). https://doi.org/10.1186/s40900-017-0062-2
